# Oral Tissues Interactions with Lights and Matters

**DOI:** 10.1155/2015/308138

**Published:** 2015-03-26

**Authors:** Samir Nammour, Umberto Romeo, Carlos de Paula Eduardo, Toni Zeinoun

**Affiliations:** ^1^Department of Dental Sciences, Faculty of Medicine, University of Liege, Liege, Belgium; ^2^Department of Oral and Maxillofacial Sciences, Sapienza University of Rome, Rome, Italy; ^3^Faculty of Dentistry, University of São Paulo (FOUSP), São Paulo, SP, Brazil; ^4^Department of Oral and Maxillofacial Surgery, Faculty of Dentistry, Lebanese University, Beirut, Lebanon

Oral tissues interactions with lights and matters are gaining interest in the medical field and more specifically in dentistry. The introduction of tooth colored resin composite had a significant impact in pushing dentistry into the esthetic arena based on 2 major developments: adhesion and light-cured materials. Different light curing devices were introduced based on the mode of action and wavelength properties. But probably one of the major breakthroughs of technology in clinical dentistry is the integration of laser therapy as a therapeutic option into treatment plan for clinical improvement. The introduction of different laser devices with different wavelengths (diode lasers, Nd:YAG, Er:YAG, Er, Cr:YSGG, and CO_2_) allows specific and selective clinical applications on soft and/or hard dental tissues opening the door to laser therapy in diagnosis, cavity preparation, esthetic and oral surgeries.

This special issue is a compendium of different studies and fundamental and clinical researches. Some papers are focused on the effect of laser therapy in dental bleaching, its side effects on enamel and pulp, and the advised chemical components and techniques. We also included interesting studies about tissue engineering and pulp revascularization of immature permanent teeth which probably will be one of the most challenging topics in the near future. Main papers treating basic and fundamental researches reported very interesting methods and results in the application of laser therapy, sonic/rotary instruments, and light cure devices in restorative and esthetic dentistry (bonding, marginal integrity, polymerization, and dental hypersensitivity) and biological interaction (stem cells, biopsy, and histology). Some papers on clinical studies encompass the effect of laser on the periodontium, effect of mouthwash on biofilm control, and the use of analgesic combination in the management of chronic temporomandibular disorders.

We hope that the content of this special issue provides valuable insights on the interaction of oral tissues with lights and matters to clinicians and researchers.

*Samir Nammour* *Samir Nammour* *Umberto Romeo* *Umberto Romeo* *Carlos de Paula Eduardo* *Carlos de Paula Eduardo* *Toni Zeinoun* *Toni Zeinoun*

